# The impact of nebulized epoprostenol and iloprost on hemoglobin oxygen affinity: an ex vivo experiment

**DOI:** 10.1152/ajplung.00084.2022

**Published:** 2022-05-03

**Authors:** Simon Woyke, Norbert Mair, Thomas Haller, Marco Ronzani, David Plunser, Herbert Oberacher, Hannes Gatterer, Christopher Rugg, Mathias Ströhle

**Affiliations:** ^1^Department of Anaesthesiology and Critical Care Medicine, Medical University of Innsbruck, Innsbruck, Austria; ^2^Institute of Physiology and Medical Physics, Medical University of Innsbruck, Innsbruck, Austria; ^3^Institute of Legal Medicine and Core Facility Metabolomics, Medical University of Innsbruck, Innsbruck, Austria; ^4^Institute of Mountain Emergency Medicine, Eurac Research, Bolzano, Italy

**Keywords:** Hill coefficient, oxygen dissociation curve, P50, prostacyclin

## Abstract

Inhalational prostacyclins act as strong vasodilators, potentially improving oxygenation by reducing shunt fraction and ventilation-perfusion mismatch. As prostacyclin receptors are known to be present on human erythrocytes, possible direct effects on hemoglobin oxygen transport were further explored by examining the sole in vitro influence of prostacyclins on hemoglobin oxygen (Hb-O_2_) affinity. Venous blood samples from 20 healthy volunteers were exposed in vitro to supramaximal doses of epoprostenol, iloprost, and compared with control. By high-throughput measurements, hemoglobin oxygen dissociation curves (ODCs) were derived. Hb-O_2_ affinity, expressed by P50 and Hill coefficient, was determined and analyzed for three subgroups: males (*n* = 10), females not taking oral contraceptives (*n* = 4), and females taking oral contraceptives (*n* = 6). Epoprostenol significantly decreased P50 in all (males, females without contraceptives, and females taking oral contraceptives) [27.5 (26.4–28.6) mmHg (control) vs. 24.2 (22.7–25.3) mmHg; *P* < 0.001. median (interquartile range, IQR)] thereby increasing Hb-O_2_ affinity. Inversely, iloprost only showed significant effects in females taking oral contraceptives where P50 was markedly increased and therefore Hb-O_2_ affinity decreased [28.4 (27.9–28.9) mmHg (control) vs. 34.4 (32.2–36.0) mmHg; *P* < 0.001]. Prostacyclin-receptor stimulation and subsequent cAMP-mediated ATP release from erythrocytes are discussed as a possible underlying mechanism for the effect of epoprostenol on Hb-O_2_ affinity. The reason for the sex hormone-modified iloprost effect remains unclear. Being aware of potentially differing effects on Hb-O_2_ affinity might help select the right prostacyclin (epoprostenol vs. iloprost) depending on the patient and the underlying disease (e.g., acute respiratory distress syndrome vs. peripheral arterial disease).

## INTRODUCTION

Being the active metabolites of arachidonic acid, prostaglandins are important mediators in inflammation and pain origination (1). Regarding small vessels, prostaglandins can potentially cause both vasodilatation (e.g., prostacyclin as prostaglandin I_2_; PGI_2_) or vasoconstriction (e.g., thromboxane A2; 2). As a medication with potent vasodilatory and platelet aggregation-inhibitory effects, prostacyclin is also known as epoprostenol (3). Due to its short half-life of only a few minutes epoprostenol is continuously administered, either intravenously or inhalational (1, 3). Epoprostenol can be used for the treatment of pulmonary arterial hypertension, acute respiratory distress syndrome (ARDS), and COVID-19 associated respiratory failure (3–5). Iloprost, a synthetic prostacyclin analog, inhibits platelet activation and acts as an arterial vasodilator thereby decreasing peripheral resistance (6). The plasma half-life is comparatively longer with 20–30 min and it can also be administered like epoprostenol (1). It has been well studied in the treatment of peripheral vascular disease and Raynaud’s phenomenon, but it is also considered in the treatment of myocardial ischemia and infarction (6). Furthermore, inhalational iloprost can successfully be applied in the treatment of pulmonary arterial hypertension (7–10). Iloprost has shown improved walking distance and long-term recovery in patients with primary pulmonary hypertension and heart failure (NYHA III-IV; 8, 10).

The hemoglobin oxygen dissociation curve (ODC) describes the binding of a maximum of four molecules of oxygen (O_2_) to hemoglobin (Hb; 11, 12). Several factors [e.g., pH, carbon dioxide, temperature, 2,3-bisphosphoglycerate (2,3-BPG), adenosine triphosphate (ATP), and more] are known to have effects on the ODC (13), whereas further in vivo effectors are suspected but still unknown (14). Moreover, a pharmacological change in Hb-O_2_ affinity, i.e., an increase in Hb-O_2_ affinity, is under debate of being potentially beneficial in patients suffering from COVID-19 associated respiratory failure (15–17). Hb-O_2_ affinity can be increased, indicated by a left-shifted ODC and a respective decrease in P50 [O_2_ partial pressure ($\text{P}{\text{O}}_{2}$) at which 50% of Hb is saturated with O_2_]; or vice versa, Hb-O_2_ affinity is decreased, indicated by a right-shifted ODC with an increase in P50. The Hill coefficient (HC) is the parameter describing the maximum steepness of the ODC’s slope in the Hill plot, thus being the parameter indicating the Hb-O_2_ binding cooperativity (11).

The data about the interaction of prostacyclins with Hb-O_2_ affinity is scarce and sometimes contradictory: in 1973 Collins et al. (18) investigated the potential effects of some prostaglandins (PGE_1_, PGE_2_, PGA_1_, PGA_2_, PGF_1α_, and PGF_2α_) on the ODC by adding prostaglandin solutions to blood samples in vitro, but could not detect any changes in P50. At the same time, Rabinowitz et al. (19) contrarily reported that PGE_2_ slightly increased Hb-O_2_ affinity in sickle erythrocytes. With regard to iloprost, a significant increase in P50 accompanied by an increase in 2,3-BPG after intravenous administration was demonstrated by Di Perri et al. (20) in 1990.

In patients receiving prostacyclins such as epoprostenol or iloprost, oxygen transport is particularly critical as they are often suffering from pulmonary arterial hypertension, ARDS, COVID-19, or peripheral vascular disease. An interaction of oral contraceptives with endovascular prostacyclin levels, e.g., suppressed prostacyclin production with prolonged use of oral contraception, has been reported before (21, 22). To eliminate the potential confounder of improving oxygenation through pulmonary vasodilation, we performed this ex vivo study to examine the sole effect on Hb-O_2_ affinity. Moreover, to investigate the effects of humidifiers, which are usually used for intubated patients, the experiment included a setup where the gas mixtures were humidified before entering the special ODC plate. To examine the possible interactions of the humidification and the nebulization of gases, e.g., absorption of nebulized particles in the humidifier, nebulizers have been placed proximal or distal to the humidifier. In this study, we investigated the in vitro effect of inhalational administered epoprostenol and iloprost. Nebulizer are used both, proximal and distal to the humidifier, thus the impact of the nebulizer’s position was investigated as a secondary outcome.

## METHODS

This study was approved by the Ethical Board of Medical University of Innsbruck (1265/2020) and is registered with clinicaltrials.gov (NCT04612270). All subjects gave a written informed consent.

Twenty healthy volunteers (10 female and 10 male) were included in this investigation. The study population consisted of 10 females and 10 males. Of them, six females were taking oral contraceptives, whereas four females were not. Subjects were between 18 and 40 yr old, nonsmokers, not pregnant or breastfeeding, had no hemoglobinopathy, or recent history of illness, blood loss, trauma, or multiday trips to high altitude (>3,000 m). Venous blood was drawn with a minimum period of stasis from an antecubital vein and immediately stored on ice. Blood gas analysis (ABL800 flex, Radiometer, Krefeld, Germany) was performed of aliquots of every heparinized blood sample. Aliquots of the whole blood samples were stored at −80°C for 2,3-BPG and ATP determination.

Complete ODCs were recorded using a novel in vitro method for high-throughput ODC measurements (23). Fifteen microliter of untreated whole blood samples are pipetted into the wells of a specially modified gas-tight 96-well plate to create a thin blood film on the well bottom of only one to three layers of red blood cells. During the measurement, these blood films are overflown by gas mixtures with an oxygen gradient from 20 vol% to 0 vol%. $\text{P}{\text{O}}_{2}$ is measured at the beginning and at the end of the gas flow through system in the special 96-well plate and $\text{S}{\text{O}}_{2}$ via dual-wavelength absorption in every single sample containing well. For details see Woyke et al. (23). A gas mixture containing a carbon dioxide partial pressure of 40 mmHg was used, temperature was set to 37°C. In the four-channel ODC plate blood samples and an internal hemoglobin standard solution (Equil QC 463, RNA medical, Devens, MA) were exposed in vitro to different gas compositions side by side, including a control gas system with a standard gas mixture (0–20 vol% O_2_, 40 mmHg $\text{P}\text{C}{\text{O}}_{2}$, and 74.7–94.7 vol% N_2_). Nebulizers (Aerogen Solo, Aerogen, Galway, Ireland) were implemented both, proximal or distal to the humidifier in the ODC method. In addition, in the control gas system, a nebulizer was integrated to guarantee equal conditions. A supramaximal dose of 10 µg (1 mL) iloprost (undiluted, Ventavis, Bayer, Leverkusen, Germany) or epoprostenol (Dynovas, Gebro Pharma, Fieberbrunn, Austria) was injected in the supply compartment of the nebulizer and nebulized exactly 5 min before the ODC measurement. Nebulization was confirmed visually by condensation on transparent surfaces of the gas system that cleared within 3 min, and 2 min before the start of the measurement. To investigate the influence of the humidifier on nebulization, the nebulizers were implemented into the experimental setting either proximal or distal to the humidifier. Triplicate measurements of every blood sample exposed to both prostacyclins, proximal or distal to the humidifier, and exposed to a control gas system without nebulization, were recorded.

Concentrations of 2,3-BPG and ATP were determined with a validated liquid chromatography-tandem mass spectrometry (LC-MS/MS) method. Isotopically labeled analogs were used as internal standards (ATP ^13^C_10_ and 2,3-BPG-^13^C_3_). See Supplemental Material: https://doi.org/10.6084/m9.figshare.19642452 for details.

Data collection, ODC curve fitting procedure, P50 and HC calculations were made using Excel (Microsoft, 2016). Statistical analysis was performed using R (v4.0.2, R Core Team, www.R-project.org) and RStudio (v1.2.5001, RStudio Inc., Boston, MA). Due to nonnormal distribution of the small sample size, nonparametric tests were applied. When comparing baseline characteristics between subgroups, Kruskal–Wallis or Mann–Whitney *U* test was applied. Shown to be robust for small sample sizes, ANOVA type statistics modified for nonparametric longitudinal data (R package: nparLD) was utilized to analyze the effects of substances with regard to controls (24). Effect size was computed via paired rank biserial correlation (R package: effect size). A *P* value < 0.05 was considered significant, data are presented as median ± interquartile range.

## RESULTS

Age was comparable between all three groups. Hb and $\text{P}\text{C}{\text{O}}_{2}$ levels were increased in males when compared with females in initial blood gas analyses (Table 1). However, before the measurements in the in vitro experiments, whole blood samples were exposed to 40 mmHg $\text{P}\text{C}{\text{O}}_{2}$ to exclude effects of the $\text{P}\text{C}{\text{O}}_{2}$ Bohr effect. P50 was increased in females when compared with males. Baseline characteristics are shown in Table 1. Concentrations of 2,3-BPG and ATP as well as HC did not differ statistically significant between groups.

**Table 1.** Baseline characteristics

	Males (*n* = 10)	Females Not Taking Oral Contraceptives (*n* = 4)	Females Taking Oral Contraceptives (*n* = 6)	*P*
Age, yr	29.5 (27.3–30.0)	29.0 (28.8–29.0)	28.5 (28.0–29.8)	0.914
Hb, g/dL	15.1 (14.8–15.8)	13.3 (13.1–13.6)	13.1 (12.6–14.0)	0.001
pH	7.34 (7.31–7.37)	7.37 (7.34–7.39)	7.38 (7.37–7.40)	0.078
$\text{P}\text{C}{\text{O}}_{2}$, mmHg	53 (45–59)	39 (38–44)	40 (39–42)	0.005
HCO_3_^−^, mmol/L	23.7 (23.5–24.6)	22.2 (21.9–22.6)	22.4 (21.8–23.4)	0.065
2,3-BPG, µg/mL	785 (747–791)	753 (739–770)	752 (725–787)	0.644
2,3-BPG, µmol/gHb	18.8 (17.5–19.7)	21.4 (20.5–22.1)	21.4 (19.3–23.6)	0.064
ATP, µg/mL	356 (347–389)	384 (368–402)	342 (331–371)	0.249
ATP, µmol/gHb	4.73 (4.51–5.09)	5.71 (5.36–6.04)	5.23 (4.97–5.37)	0.053
P50, mmHg	26.3 (24.7–27.5)	28.3 (27.2–29.5)	28.4 (27.9–28.9)	0.030
HC	2.76 (2.70–2.78)	2.84 (2.78–3.00)	2.76 (2.73–2.81)	0.456

Values are median (interquartile range). *P* refers to overall comparison of all three subgroups (Kruskal–Wallis test). ATP, adenosine triphosphate concentration; Hb, hemoglobin concentration; HC, hill coefficient; HCO_3_^−^, bicarbonate concentration; $\text{P}\text{C}{\text{O}}_{2}$, carbon dioxide partial pressure; P50, oxygen partial pressure of Hb half-saturation; 2,3-BPG, 2,3-bisphosphoglycerate concentration.

**Figure 1.**
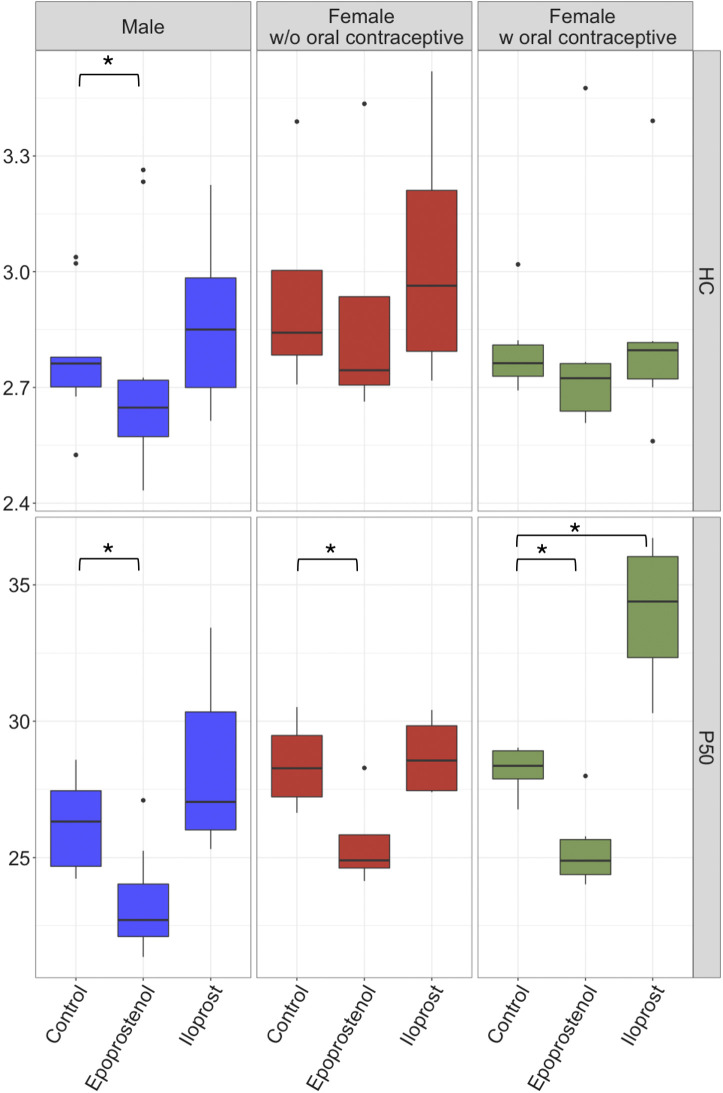
Box plots of P50 and HC. Blood samples, divided into three subgroups (male *n* = 10; female without oral contraceptive *n* = 6; female with oral contraceptive *n* = 4), were exposed to supramaximal doses of epoprostenol (10 µg) or iloprost (10 µg), and controls. Nebulizers were placed distal to the humidifiers. Brackets with * indicate significant group differences, analyzed with ANOVA type statistics modified for nonparametric longitudinal data. HC, Hill coefficient; P50, oxygen partial pressure of Hb half-saturation (mmHg).

In males and females regardless of contraception, epoprostenol significantly decreased P50 when compared with controls (*P* < 0.001; Fig. 1, Table 2). Inversely to this epoprostenol-induced left shift of the ODC. There was a right-shift of the ODC due to iloprost exposure only in blood samples from females taking oral contraceptives (*P* < 0.001; Fig. 1, Table 2). Besides a significant decrease of HC in males exposed to epoprostenol [2.76 (2.70–2.78) vs. 2.65 (2.57–2.72); *P* = 0.019], HCs were not influenced by epoprostenol or iloprost, in spite of changes in P50.

**Table 2.** P50 results for the three subgroups

	Males (*n* = 10)	Females Not Taking Oral Contraceptives (*n* = 4)	Females Taking Oral Contraceptives (*n* = 6)
Control	26.3 (24.7–27.4)	28.3 (27.2–29.5)	28.4 (27.9–28.9)
Epoprostenol			
Nebulizer distal to the humidifier	22.7 (22.1–24.0) *P* < 0.001	24.9 (24.6–25.8) *P* < 0.001	24.9 (24.4–25.7) *P* < 0.001
Epoprostenol			
Nebulizer proximal to the humidifier	24.3 (23.0–25.2) *P* < 0.001	26.7 (26.3–27.1) *P* = 0.035	27.2 (26.5–27.8) *P* < 0.001
Iloprost			
Nebulizer distal to the humidifier	27.0 (26.0–30.3) *P* = 0.167	28.6 (27.5–29.8) *P* = 0.796	34.4 (32.3–36.0) *P* < 0.001
Iloprost			
Nebulizer proximal to the humidifier	25.7 (24.5–28.0) *P* = 1.000	27.1 (25.0–29.0) *P* = 0.417	33.5 (30.2–34.4) *P* = 0.040

P50 (in mmHg) results for the three subgroups shown for epoprostenol and iloprost in relation to nebulizer position [median (interquartile range)]. *P* refers to comparison with control; ANOVA type statistics modified for nonparametric longitudinal data. P50, oxygen partial pressure of Hb half-saturation.

As a secondary end point, the nebulizers were implemented both, proximal or distal to the humidifier in the experimental setup. As seen in Table 2, the substance-induced changes in Hb-O_2_ affinity were less pronounced when the nebulizer was implemented proximal to the humidifier. Therefore, all aforementioned results (e.g., Fig. 1) refer to the constellation nebulizer distal to the humidifier.

A significantly increased P50 in all females when compared with males was present not only in controls [28.4 (27.5–29.0) vs. 26.3 (24.7–27.5); *P* = 0.007] but also in epoprostenol [24.9 (24.4–25.7) vs. 22.7 (22.1–24.0); *P* = 0.007] and iloprost [31.1 (29.8–34.5) vs. 27.0 (26.0–30.3); *P* = 0.023] measurements.

## DISCUSSION

The effects of epoprostenol and iloprost on Hb-O_2_ affinity differed substantially. While epoprostenol increased Hb-O_2_ affinity in all subgroups, iloprost decreased Hb-O_2_ affinity in females taking oral contraceptives and had no effects in the other subgroups. The most obvious underlying pathomechanism would be a stimulation of present prostacyclin receptors on human erythrocytes (25). Activation leads to an increase in intracellular cAMP itself leading to an increased release of ATP (25–27). As organic phosphates such as 2,3-BPG or ATP decrease Hb-O_2_ affinity (12, 28), a release of ATP with therefore subsequently decreased intracellular levels will increase Hb-O_2_ affinity. However, the pathophysiological mechanism underlying the sex hormone modified iloprost-induced right-shift of the ODC remains unclear. Nevertheless, this finding is of great interest, as contraception is recommended for women taking iloprost due to teratogenic toxicity. Despite changes in Hb-O_2_ affinity, the cooperativity of O_2_ binding to Hb, i.e., the HC, was not affected by both prostacyclins.

By describing the functionality of hemoglobin oxygen transport, Hb-O_2_ affinity has a strong impact on both, oxygen uptake in the lungs and oxygen unloading at the tissue level. In patients, epoprostenol and iloprost are primarily used because of their vasodilatory effect, particularly in the pulmonary arterial system. However, the underlying mechanisms of pulmonary arterial hypertension vary widely. In ARDS, among other problems, pulmonary oxygenation is at risk, commonly expressed by the Horovitz index. An increase in Hb-O_2_ affinity, indicated by a left-shifted ODC, can potentially improve complete oxygenation of Hb during the short passage time of erythrocytes through the lungs and mitigate hypoxemia. In particular, when applied inhalational, the combination of local vasodilation with an increase in Hb-O_2_ affinity might improve pulmonary oxygen uptake and thus ameliorate hypoxemia in patients with COVID-19 (15). On the other hand, in myocardial ischemia/infarction, peripheral vascular disease or the Raynaud’s phenomenon, an increased Hb-O_2_ affinity might aggravate poor oxygenation due to constrained Hb-O_2_ unloading in the tissue at risk (e.g., myocardium, peripheral soft tissue). Kleen and Zwissler (29) stated that the use of inhalational prostacyclins might be beneficial in specific situations during surgery (e.g., single lung ventilation, lung transplantation, liver transplantation, cardiac surgery, and sickle cell anemia) where modifying Hb-O_2_ affinity, accompanied by the effect of vasodilation, might ameliorate oxygenation. Having two prostacyclins with comparable efficacy and safety characteristics (1), the effect on Hb-O_2_ affinity might become an additional parameter supporting the selection of either epoprostenol or iloprost based on the underlying pathophysiological mechanism. Taken together, the present data suggest that in conditions where patients can potentially profit from ameliorated oxygenation at the lung level, epoprostenol might be the drug of choice. Whereas in conditions of enhanced peripheral oxygen need iloprost might be preferred. Certainly, these assumptions have to be confirmed in the clinical setting before final recommendations can be given.

Iloprost is associated with a decrease in Hb-O_2_ affinity in females taking oral contraceptives. Females in comparison with males had lower baseline Hb-O_2_ affinity along with lower Hb concentration, which is consistent with common knowledge (30, 31). While chronical estrogen uptake (i.e., oral contraceptives) is able to change prostacyclin levels in vivo (21, 22), the mechanism of how estrogen interferes with the iloprost effect is obscure. However, early reports indicate that estrogen itself may influence Hb-O_2_ affinity (30). There are further studies needed to investigate the effect of sex hormones on Hb-O_2_ affinity and the underlying mechanisms in a larger group. During the exposure to nebulized prostacyclins and the ODC determination blood samples were constantly flushed by a $\text{P}\text{C}{\text{O}}_{2}$ of 40 mmHg. Due to the high solubility of $\text{P}\text{C}{\text{O}}_{2}$ in liquids, the difference in $\text{P}\text{C}{\text{O}}_{2}$ between males and females measured via blood gas analysis should be compensated.

Up to now, there is no consensus on whether a nebulizer should be combined with a humidifier, and if the humidifier should be placed between nebulizer and patient or the other way around (32, 33). Therefore, the effect of epoprostenol and iloprost was measured in relation to the nebulizer position. Our data suggest placing the nebulizer between humidifier and patient, if a humidifier is used, to optimize the effect of prostacyclins on Hb-O_2_ affinity and probably also on vasodilation. Humidifier’s water might absorb nebulized prostacyclins and reduce the concentration reaching the patient.

### Limitations

Due to the experimental setting and the ex vivo design of this study, a dose dependency could not be investigated as the prostacyclins were applied under idealized conditions potentially resulting in supramaximal dosing. Furthermore, complex pharmacokinetic aspects were not considered. In this experimental setting, in which blood samples were directly exposed to nebulized prostacyclins, one must suppose that only the situation in pulmonary vessels, directly linked to alveoli, is displayed. Furthermore, the mechanisms leading to changes in Hb-O_2_ affinity could not be determined and further investigations are needed to detect the underlying (patho)physiological mechanisms. Importantly, the composition and variety of oral contraceptives taken are unknown, as is any information on the menstrual cycle and actual plasma concentrations of sex hormone. Dividing subjects into three subgroups resulted in a small samples size requiring nonparametric testing. Despite a large effect size suggesting real and major effects of prostacyclins on the ODC, these results need to be confirmed by larger sample sizes (Table 3).

**Table 3.** Effect size for the three subgroups

	Males (*n* = 10) *P* Effect Size (95% CI)	Females Not Taking Oral Contraceptives(*n* = 4) *P* Effect Size (95% CI)	Females Taking Oral Contraceptives (*n* = 6) *P* Effect Size (95% CI)
Epoprostenol			
Nebulizer distal to the humidifier	*P* < 0.001 −1.00 (−1.00, −1.00)	*P* < 0.001 −1.00 (−1.00, −1.00)	*P* < 0.001 −1.00 (−1.00, −1.00)
Epoprostenol			
Nebulizer proximal to the humidifier	*P* < 0.001 −1.00 (−1.00, −1.00)	*P* = 0.035 −1.00 (−1.00, −1.00)	*P* < 0.001 −1.00 (−1.00, −1.00)
Iloprost			
Nebulizer distal to the humidifier	*P* = 0.167 0.53 (−0.11, 0.86)	*P* = 0.796 0.00 (−0.79, 0.79)	*P* < 0.001 1.00 (1.00, 1.00)
Iloprost			
Nebulizer proximal to the humidifier	*P* = 1.000 −0.05 (−0.64, 0.57)	*P* = 0.417 −0.40 (−0.90, 0.57)	*P* = 0.040 0.81 (0.23, 0.97)

Effect size for the three subgroups shown for epoprostenol and iloprost in relation to nebulizer position (*P* refers to comparison with control; paired rank biserial correlation). CI, confidence interval.

Due to the small sample volume of blood (15 µL), which was plated to films (23), 2,3-BPG and ATP could only be measured in controls and not after the exposure with prostacyclins. Values of 2,3-BPG and ATP are slightly higher than those reported in the literature. The rather long storage time (i.e., 12 mo) at −80°C might be the reason for that since storage time leads to elevated 2,3-BPG and ATP levels (34). Nonetheless, comparability between the groups in the present study should be valid because the storage time of all samples was exactly the same. To increase solubility, epoprostenol is diluted and applied in a solution with a pH of 10.5. In control experiments carried out on the blood of one subject only, there was no relevant pH effect in the blood film exposed to nebulized epoprostenol (data not shown). Nonetheless, a possible influence of the overflowing alkaline solution on the blood film’s pH cannot be completely excluded.

### Conclusions

Epoprostenol and iloprost affect Hb-O_2_ affinity in opposite ways. Epoprostenol leads to an increase in Hb-O_2_ affinity in all subgroups, whereas iloprost leads to a decrease only in females taking oral contraceptives. This difference in the effect on Hb-O_2_ affinity might enable clinicians to include knowledge about Hb-O_2_ affinity into their therapeutic considerations when choosing the right inhalational prostacyclin for the right patient, depending on the underlying pathophysiology.

## DATA AVAILABILITY

Data will be made available by the corresponding author upon reasonable request.

## SUPPLEMENTAL DATA

10.6084/m9.figshare.19642452 Supplemental Material: https://doi.org/10.6084/m9.figshare.19642452.

## GRANTS

This study was funded by equity capital.

## DISCLOSURES

No conflicts of interest, financial or otherwise, are declared by the authors.

## AUTHOR CONTRIBUTIONS

S.W., N.M., T.H., M.R., H.G., C.R., and M.S. conceived and designed research; S.W., N.M., T.H., M.R., D.P., H.O., and M.S. performed experiments; S.W., N.M., D.P., H.O., and C.R. analyzed data; S.W., N.M., T.H., M.R., H.G., C.R., and M.S. interpreted results of experiments; C.R. prepared figures; S.W. drafted manuscript; S.W., N.M., T.H., M.R., D.P., H.O., H.G., C.R., and M.S. edited and revised manuscript; S.W., N.M., T.H., M.R., D.P., H.O., H.G., C.R., and M.S. approved final version of manuscript.
